# Solving unsolved rare neurological diseases—a Solve-RD viewpoint

**DOI:** 10.1038/s41431-021-00901-1

**Published:** 2021-05-10

**Authors:** Rebecca Schüle, Dagmar Timmann, Corrie E. Erasmus, Jennifer Reichbauer, Melanie Wayand, Solve-RD-DITF-RND, Jonathan Baets, Peter Balicza, Patrick Chinnery, Alexandra Dürr, Tobias Haack, Holger Hengel, Rita Horvath, Henry Houlden, Erik-Jan Kamsteeg, Christoph Kamsteeg, Katja Lohmann, Alfons Macaya, Anna Marcé-Grau, Ales Maver, Judit Molnar, Alexander Münchau, Borut Peterlin, Olaf Riess, Ludger Schöls, Rebecca Schüle, Giovanni Stevanin, Matthis Synofzik, Vincent Timmerman, Bart van de Warrenburg, Nienke van Os, Jana Vandrovcova, Melanie Wayand, Carlo Wilke, Bart van de Warrenburg, Ludger Schöls, Carlo Wilke, Andrea Bevot, Stephan Zuchner, Sergi Beltran, Steven Laurie, Leslie Matalonga, Holm Graessner, Matthis Synofzik, The Solve-RD Consortium, Holm Graessner, Birte Zurek, Kornelia Ellwanger, Stephan Ossowski, German Demidov, Marc Sturm, Julia M. Schulze-Hentrich, Peter Heutink, Han Brunner, Hans Scheffer, Nicoline Hoogerbrugge, Alexander Hoischen, Peter A. C. ’t Hoen, Lisenka E. L. M. Vissers, Christian Gilissen, Wouter Steyaert, Karolis Sablauskas, Richarda M. de Voer, Erik Janssen, Elke de Boer, Marloes Steehouwer, Burcu Yaldiz, Tjitske Kleefstra, Anthony J. Brookes, Colin Veal, Spencer Gibson, Marc Wadsley, Mehdi Mehtarizadeh, Umar Riaz, Greg Warren, Farid Yavari Dizjikan, Thomas Shorter, Ana Töpf, Volker Straub, Chiara Marini Bettolo, Sabine Specht, Jill Clayton-Smith, Siddharth Banka, Elizabeth Alexander, Adam Jackson, Laurence Faivre, Christel Thauvin, Antonio Vitobello, Anne-Sophie Denommé-Pichon, Yannis Duffourd, Emilie Tisserant, Ange-Line Bruel, Christine Peyron, Aurore Pélissier, Sergi Beltran, Ivo Glynne Gut, Steven Laurie, Davide Piscia, Leslie Matalonga, Anastasios Papakonstantinou, Gemma Bullich, Alberto Corvo, Carles Garcia, Marcos Fernandez-Callejo, Carles Hernández, Daniel Picó, Ida Paramonov, Hanns Lochmüller, Gulcin Gumus, Virginie Bros-Facer, Ana Rath, Marc Hanauer, Annie Olry, David Lagorce, Svitlana Havrylenko, Katia Izem, Fanny Rigour, Alexandra Durr, Claire-Sophie Davoine, Léna Guillot-Noel, Anna Heinzmann, Giulia Coarelli, Gisèle Bonne, Teresinha Evangelista, Valérie Allamand, Isabelle Nelson, Rabah Ben Yaou, Corinne Metay, Bruno Eymard, Enzo Cohen, Antonio Atalaia, Tanya Stojkovic, Milan Macek, Marek Turnovec, Dana Thomasová, Radka Pourová Kremliková, Vera Franková, Markéta Havlovicová, Vlastimil Kremlik, Helen Parkinson, Thomas Keane, Dylan Spalding, Alexander Senf, Peter Robinson, Daniel Danis, Glenn Robert, Alessia Costa, Christine Patch, Mike Hanna, Henry Houlden, Mary Reilly, Jana Vandrovcova, Francesco Muntoni, Irina Zaharieva, Anna Sarkozy, Peter de Jonghe, Vincenzo Nigro, Sandro Banfi, Annalaura Torella, Francesco Musacchia, Giulio Piluso, Alessandra Ferlini, Rita Selvatici, Rachele Rossi, Marcella Neri, Stefan Aretz, Isabel Spier, Anna Katharina Sommer, Sophia Peters, Carla Oliveira, Jose Garcia Pelaez, Ana Rita Matos, Celina São José, Marta Ferreira, Irene Gullo, Susana Fernandes, Luzia Garrido, Pedro Ferreira, Fátima Carneiro, Morris A. Swertz, Lennart Johansson, Joeri K. van der Velde, Gerben van der Vries, Pieter B. Neerincx, Dieuwke Roelofs-Prins, Sebastian Köhler, Alison Metcalfe, Alain Verloes, Séverine Drunat, Caroline Rooryck, Aurelien Trimouille, Raffaele Castello, Manuela Morleo, Michele Pinelli, Alessandra Varavallo, Manuel Posada De la Paz, Eva Bermejo Sánchez, Estrella López Martín, Beatriz Martínez Delgado, F. Javier Alonso García de la Rosa, Andrea Ciolfi, Bruno Dallapiccola, Simone Pizzi, Francesca Clementina Radio, Marco Tartaglia, Alessandra Renieri, Elisa Benetti, Peter Balicza, Maria Judit Molnar, Ales Maver, Borut Peterlin, Alexander Münchau, Katja Lohmann, Rebecca Herzog, Martje Pauly, Alfons Macaya, Anna Marcé-Grau, Andres Nascimiento Osorio, Daniel Natera de Benito, Hanns Lochmüller, Rachel Thompson, Kiran Polavarapu, David Beeson, Judith Cossins, Pedro M. Rodriguez Cruz, Peter Hackman, Mridul Johari, Marco Savarese, Bjarne Udd, Rita Horvath, Gabriel Capella, Laura Valle, Elke Holinski-Feder, Andreas Laner, Verena Steinke-Lange, Evelin Schröck, Andreas Rump

**Affiliations:** 1 Hertie Institute for Clinical Brain Research (HIH), Center of Neurology, University of Tübingen, Tübingen, Germany; 2 German Center for Neurodegenerative Diseases (DZNE), University of Tübingen, Tübingen, Germany; 3 European Reference Network for Rare Neurological Diseases, Tübingen, Germany; 4 Department of Neurology and Center for Translational Neuro- and Behavioral Sciences (C-TNBS), University Hospital Essen, Essen, Germany; 5 Department of Pediatric Neurology, Radboud University Medical Center, Amalia Children’s Hospital, Donders Institute for Brain, Cognition and Behavior, Nijmegen, The Netherlands; 6 Department of Neurology, Donders Centre for Brain, Cognition and Behavior, Radboud University Medical Center, Nijmegen, The Netherlands; 7 Department of Pediatric Neurology and Developmental Medicine, University Children’s Hospital, Tübingen, Germany; 8 Dr. John T. Macdonald Foundation Department of Human Genetics, John P. Hussman Institute for Human Genomics, University of Miami Miller School of Medicine, Miami, FL USA; 9 CNAG-CRG, Centre for Genomic Regulation (CRG), Barcelona Institute of Science and Technology (BIST), Barcelona, Spain; 10 Universitat Pompeu Fabra (UPF), Barcelona, Spain; 11 Facultat de Biologia, Departament de Genètica, Microbiologia i Estadística, Universitat de Barcelona (UB), Barcelona, Spain; 12 Institute of Medical Genetics and Applied Genomics, University of Tübingen, Tübingen, Germany; 13 Peripheral Neuropathy Research Group, University of Antwerp, Antwerp, Belgium; 14 Neuromuscular Reference Centre, Department of Neurology, Antwerp University Hospital, Antwerpen, Belgium; 15 Laboratory of Neuromuscular Pathology, Institute Born-Bunge, University of Antwerp, Antwerpen, Belgium; 16 Semelweis University Budapest, Budapest, Hungary; 17 Center for Hereditary Tumor Syndromes, University Hospital Bonn, Bonn, Germany; 18 Institut National de la Santé et de la Recherche Medicale (INSERM) U1127, Paris, France; 19 Centre National de la Recherche Scientifique, Unité Mixte de Recherche (UMR), Paris, France; 20 Unité Mixte de Recherche en Santé 1127, Université Pierre et Marie Curie (Paris 06), Sorbonne Universités, Paris, France; 21 Department of Neurodegeneration, Hertie Institute for Clinical Brain Research (HIH), University of Tübingen, Tübingen, Germany; 22 University of Cambridge, England, UK; 23 Department of Neuromuscular Diseases, UCL Queen Square Institute of Neurology and The National Hospital for Neurology and Neurosurgery, London, UK; 24 Department of Human Genetics, Radboud University Medical Center, Nijmegen, The Netherlands; 25 University of Lübeck, Lübeck, Germany; 26 Hospital Vall d’Hebron, Barcelona, Spain; 27 University of Ljubljana, Ljubljana, Slovenia; 28 Centre for Rare Diseases, University of Tübingen, Tübingen, Germany; 29 Institut du Cerveau-ICM, Paris, France; 30 Ecole Pratique des Hautes Etudes, Paris Sciences et Lettres Research University, Paris, France; 31 Peripheral Neuropathy Research Group, Department of Biomedical Sciences, University of Antwerp, Antwerp, Belgium; 32 Institute Born Bunge, Antwerp, Belgium; 33 Donders Institute for Brain, Cognition and Behaviour, Radboud University Medical Center, Nijmegen, The Netherlands; 34 Department of Neurology, Radboud University Medical Center, Nijmegen, The Netherlands; 35 Department of Clinical Genetics, Maastricht University Medical Centre, Maastricht, The Netherlands; 36 Radboud Institute for Molecular Life Sciences, Nijmegen, The Netherlands; 37 Department of Internal Medicine and Radboud Center for Infectious Diseases (RCI), Radboud University Medical Center, Nijmegen, The Netherlands; 38 Center for Molecular and Biomolecular Informatics, Radboud University Medical Center, Nijmegen, The Netherlands; 39 Department of Genetics and Genome Biology, University of Leicester, Leicester, UK; 40 John Walton Muscular Dystrophy Research Centre, Translational and Clinical Research Institute, Newcastle University and Newcastle Hospitals NHS Foundation Trust, Newcastle upon Tyne, UK; 41 Division of Evolution and Genomic Sciences, School of Biological Sciences, Faculty of Biology, Medicine and Health, University of Manchester, Manchester, UK; 42 Manchester Centre for Genomic Medicine, St Mary’s Hospital, Manchester University Hospitals NHS Foundation Trust, Health Innovation Manchester, Manchester, UK; 43 Dijon University Hospital, Genetics Department, Dijon, France; 44 Dijon University Hospital, Centre of Reference for Rare Diseases: Development disorders and malformation syndromes, Dijon, France; 45 Inserm - University of Burgundy-Franche Comté UMR1231 GAD, Dijon, France; 46 Dijon University Hospital, FHU-TRANSLAD, Dijon, France; 47 Dijon University Hospital, GIMI Institute, Dijon, France; 48 University of Burgundy-Franche Comté, Dijon Economics Laboratory, Dijon, France; 49 University of Burgundy-Franche Comté, FHU-TRANSLAD, Dijon, France; 50 EURORDIS-Rare Diseases Europe, Sant Antoni Maria Claret 167, Barcelona, Spain; 51 EURORDIS-Rare Diseases Europe, Plateforme Maladies Rares, Paris, France; 52 INSERM, US14 - Orphanet, Plateforme Maladies Rares, Paris, France; 53 Centre de Référence de Neurogénétique, Hôpital de la Pitié-Salpêtrière, Assistance Publique-Hôpitaux de Paris (AP-HP), Paris, France; 54 Hôpital de la Pitié-Salpêtrière, Assistance Publique-Hôpitaux de Paris (AP-HP), Paris, France; 55 Sorbonne Université, INSERM UMRS_974, Center of Research in Myology, Paris, France; 56 AP-HP, Centre de Référence de Pathologie Neuromusculaire Nord, Est, Ile-de-France, Institut de Myologie, G.H. Pitié-Salpêtrière, Paris, France; 57 Institut de Myologie, Equipe Bases de données, G.H. Pitié-Salpêtrière, Paris, France; 58 AP-HP, Unité Fonctionnelle de Cardiogénétique et Myogénétique Moléculaire et Cellulaire, G.H. Pitié-Salpêtrière, Paris, France; 59 Department of Biology and Medical Genetics, Charles University Prague-2nd Faculty of Medicine and University Hospital Motol, Prague, Czech Republic; 60 European Bioinformatics Institute, European Molecular Biology Laboratory, Wellcome Genome Campus, Hinxton, Cambridge, UK; 61 Jackson Laboratory for Genomic Medicine, Farmington, CT USA; 62 Florence Nightingale Faculty of Nursing and Midwifery, King’s College, London, UK; 63 Genetic Counselling, Genomics England, Queen Mary University of London, Dawson Hall, London, UK; 64 MRC Centre for Neuromuscular Diseases and National Hospital for Neurology and Neurosurgery, UCL Queen Square Institute of Neurology, London, UK; 65 Department of Neuromuscular Diseases, UCL Queen Square Institute of Neurology, London, UK; 66 Dubowitz Neuromuscular Centre, UCL Great Ormond Street Hospital, London, UK; 67 NIHR Great Ormond Street Hospital Biomedical Research Centre, London, UK; 68 Dipartimento di Medicina di Precisione, Università degli Studi della Campania “Luigi Vanvitelli”, Napoli, Italy; 69 Telethon Institute of Genetics and Medicine, Pozzuoli, Italy; 70 Unit of Medical Genetics, Department of Medical Sciences, University of Ferrara, Ferrara, Italy; 71 Institute of Human Genetics, University of Bonn, Bonn, Germany; 72 i3S - Instituto de Investigação e Inovação em Saúde, Universidade do Porto, Porto, Portugal; 73 IPATIMUP - Institute of Molecular Pathology and Immunology of the University of Porto, Porto, Portugal; 74 Department of Pathology, Faculty of Medicine, University of Porto, Porto, Portugal; 75 Department of Genetics, Faculty of Medicine, University of Porto, Porto, Portugal; 76 CHUSJ, Centro Hospitalar e Universitário de São João, Porto, Portugal; 77 Faculty of Sciences, University of Porto, Porto, Portugal; 78 Department of Genetics, Genomics Coordination Center, University Medical Center Groningen, University of Groningen, Groningen, The Netherlands; 79 NeuroCure Cluster of Excellence, Charité Universitätsklinikum, Charitéplatz 1, Berlin, Germany; 80 College of Health, Well-being and Life-Sciences, Sheffield Hallam University, Sheffield, UK; 81 Department of Genetics, Assistance Publique-Hôpitaux de Paris - Université de Paris, Robert DEBRE University Hospital, 48 bd SERURIER, Paris, France; 82 INSERM UMR 1141 “NeuroDiderot”, Hôpital R DEBRE, Paris, France; 83 University of Bordeaux, MRGM INSERM U1211, CHU de Bordeaux, Service de Génétique Médicale, Bordeaux, France; 84 Laboratoire de Génétique Moléculaire, Service de Génétique Médicale, CHU Bordeaux – Hôpital Pellegrin, Place Amélie Raba Léon, Bordeaux Cedex, France; 85 Institute of Rare Diseases Research, Spanish Undiagnosed Rare Diseases Cases Program (SpainUDP) & Undiagnosed Diseases Network International (UDNI), Instituto de Salud Carlos III, Madrid, Spain; 86 Genetics and Rare Diseases Research Division, Ospedale Pediatrico Bambino Gesù, IRCCS, Rome, Italy; 87 Med Biotech Hub and Competence Center, Department of Medical Biotechnologies, University of Siena, Siena, Italy; 88 Medical Genetics, University of Siena, Siena, Italy; 89 Genetica Medica, Azienda Ospedaliero-Universitaria Senese, Siena, Italy; 90 Institute of Genomic Medicine and Rare Diseases, Semmelweis University, Budapest, Hungary; 91 Clinical Institute of Genomic Medicine, University Medical Centre Ljubljana, Ljubljana, Slovenia; 92 Institute of Neurogenetics, University of Lübeck, Lübeck, Germany; 93 Neurology Research Group, Vall d’Hebron Research Institute, Universitat Autònoma de Barcelona, Barcelona, Spain; 94 Neuromuscular Disorders Unit, Department of Pediatric Neurology. Hospital Sant Joan de Déu, Barcelona, Spain; 95 Department of Neuropediatrics and Muscle Disorders, Medical Center, Faculty of Medicine, University of Freiburg, Freiburg, Germany; 96 Centro Nacional de Análisis Genómico (CNAG-CRG), Center for Genomic Regulation, Barcelona Institute of Science and Technology (BIST), Barcelona, Spain; 97 Children’s Hospital of Eastern Ontario Research Institute, University of Ottawa, Ottawa, ON Canada; 98 Nuffield Department of Clinical Neurosciences, University of Oxford, Oxford, UK; 99 Folkhälsan Research Centre and Medicum, University of Helsinki, Helsinki, Finland; 100 Tampere Neuromuscular Center, Tampere, Finland; 101 Vasa Central Hospital, Vaasa, Finland; 102 Department of Clinical Neurosciences, University of Cambridge, Cambridge, UK; 103 Bellvitge Biomedical Research Institute (IDIBELL), Barcelona, Spain; 104 Medical Genetics Center (MGZ), Munich, Germany; 105 Institute for Clinical Genetics, Faculty of Medicine Carl Gustav Carus, Technical University Dresden, Dresden, Germany; 106 Center for Personalized Oncology, University Hospital Carl Gustav Carus, Technical University Dresden, Dresden, Germany

**Keywords:** Neurodevelopmental disorders, Neurodegenerative diseases, Movement disorders

## Introduction

Rare genetic neurological disorders (RND; ORPHA:71859) are a heterogeneous group of disorders comprising >1700 distinct genetic disease entities. However, genetic discoveries have not yet translated into dramatic increases of diagnostic yield and indeed rates of molecular genetic diagnoses have been stuck at about 30–50% across NGS modalities and RND phenotypes [1, 2]. Existence of yet unknown disease genes as well as shortcomings of commonly employed NGS technologies and analysis pipelines in detecting certain variant types are typically cited to explain the low diagnosis rates.

To increase the diagnostic yield in RNDs - one of the four focus disease groups in Solve-RD - we follow two major approaches, that we will here present and exemplify: (i) systematic state-of the art re-analysis of large cohorts of unsolved whole-exome/genome sequencing (WES/WGS) RND datasets; and (ii) novel-omics approaches. Based on the way Solve-RD systematically organizes researchers’ expertise to channel this approach [3], the European Reference Network for Rare Neurological Diseases (ERN-RND) has established its own Data Interpretation Task Force (DITF) within SOLVE-RD, which is currently composed of clinical and genetic experts from 29 sites in 15 European countries.

## Systematic re-analysis of coding variation

Unsolved WES datasets (fastq) from 2048 families with RNDs were submitted by clinical sites of ERN-RND [4] to the RD-Connect Genome-Phenome Analysis Platform. Genomic data were processed and filtered as detailed [5]. The Solve-RD SNV/Indel working group reported back 74,456 variants in 2246 individuals, which were ranked according to their likelihood of being causative. One thousand nine hundred and forty-three variants in 1155 individuals (average 1.68 variants/individuum) were classified as rank 1 (genotype matches OMIM and variant (likely) pathogenic according to ACMG).

**Fig. 1:**
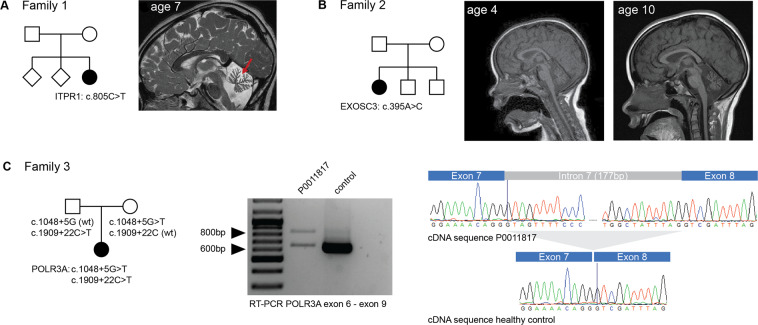
Clinical information and functional variant validation for families 1–3. **A** Pedigrees and cranial MRI of patient 1 (*NM_001168272.1(ITPR1):c.805C*>*T, p.(Arg269Trp))*. Mid-sagital MRI (T2) shows marked cerebellar atrophy at age 7. **B** Pedigree and longitudinal MRIs taken from patient 2 (pontocerebellar atrophy—*NM_016042.3(EXOSC3):c.395A*>*C, p.(Asp132Ala)*). MRIs demonstrate marked cerebellar atrophy while brainstem volume is not affected. **C** Pedigree, segregation analysis and functional analysis in family 3. The index cases carries two intronic *POLR3A* variants. Variant c.1048+5G>T is located in intron 7; RT-PCR with primers binding to sequences in exon (forward) and exon 9 (reverse) demonstrate presence of an aberrant transcript that is absent in controls. Specific amplification of this additional band and sequencing revealed that all 177 bp of intron 7 are included in the transcript. A nonsense codon in intron 7 presumably leads to termination of translation (p.Phe352_Arg353ins(23)Ter). The variant c.1909+22C>T has previously been demonstrated to lead to inclusion of the first 19 nucleotides from intron 14 into the final transcript und consequently to shift of the reading frame [8].

Based on these results and the work of the RND DITF 44 cases could be solved by this systematic re-analysis approach, which equals 29% of the re-analysed cases for which feedback was available. Reasons for solving cases were firstly updates of the respective ClinVar entry of identified variants between the time of the initial genetic workup and the Solve-RD re-analysis due to now additional available evidence. One example is the re-classification of variants in highly variably genes like *ITPR1* between 2016 and 2020 [6] (Fig. 1A).

Second, use of human phenotype ontology-based phenotypes [7] rather than diagnostic categories as well as consideration of variant-specific rather than gene-specific phenotypes enabled detection of functionally relevant variants because initial analysis focused on disease-specific panels. Mis-classification of phenotypes in RNDs is a common problem due to the considerable overlap between diagnostic categories especially in phenotypes affecting more than one neurological system. This approach i.e. allowed identification of a causative variant in *EXOSC3* (c.395A>C) that is typically associated with a ‘milder’ clinical disease course and lacking the hallmark pontine atrophy characteristic for EXOSC3-associated disease (Fig. 1B).

## Analysis of non-coding variation

The relative contribution of non-coding variation to RNDs has not been established yet and will be systematically explored by Solve-RD by combining WGS and RNA Seq. We will evaluate the added value of RNA Seq in early onset sporadic cases (Trio-WGS), multiplex recessive and dominant families.

In the meantime, the exon–intron boundaries commonly covered by WES already allow at least a glimpse into the realm of non-coding variants. Indeed, the systematic Solve-RD re-analysis top-listed a single heterozygous intronic variant in the *POLR3A* gene (*NM_007055.3(POLR3A):c.1909T*>*A: c.1909*+*22G*>A, p.Tyr637Cysfs*14) that had recently been shown to be a frequent cause of spastic ataxia [8] in trans with a second loss-of-function *POLR3A* variant in an unsolved adult patient with a spastic ataxia phenotype. No second coding *POLR3A* variant was identified. However, a variant in intron 7 of the *POLR3A* gene was discovered in the WES data (*NM_007055.3(POLR3A)*: c.1048+5G>T). RT-PCR from whole blood revealed an aberrant transcript that was absent in controls. Specific amplification and sequencing demonstrated the inclusion of all 177 bp of intron 7 into the final mRNA transcript. On protein level, this change is predicted to insert 23 amino acids coded by intron 7, followed by a stop codon (p.Phe352_Arg353ins(23)Ter) (Fig. 1C).

## Finding novel variations through novel omics

Scientific rationale drives application of novel-omics technologies in Solve-RD. From the large variety of different omics technologies that will be used by SOLVE-RD, we here present the example of long-range WGS for ataxias, which has just been initiated. For ataxias >25% of all autosomal-dominant and >50% of all autosomal-recessive ataxia patients remain unsolved despite advanced WES analysis [9]. Ataxias are unique in so far as repeat expansions represent the most frequent disease cause. Seventy-five percent of all known autosomal-dominant ataxia cases and 50% of all known autosomal-recessive ataxia cases are caused by repeat expansions [10]. We thus hypothesize that a substantial share of repeat-expansion disorders is still to be found in the large share of still unsolved WES-negative ataxia cases. Therefore, in Solve-RD we will be using long-range WGS in family ‘triplets’ from autosomal-dominant ataxia families, which will be stringently enriched for novel repeat-expansion disorders: namely only families negative not only on WES and frequent SCA repeats, but also on short-read WGS and for which DNA from >2 affected and >2 non-affected family members are available. In a first round of submission, 20 families with 44 ‘slots’ have been submitted and we are awaiting data in 2021.

## Conclusion

This viewpoint presents and exemplifies the approach being taken by Solve-RD to diagnostically solve unsolved RND. While re-analysis so far succeeded in 29% of cases, scientifically rational ‘beyond the exome’ approaches are being implemented to further unravel new RND causing genes.

## Supplementary information

Solve-RD Data Interpretation Taskforce ERN-RND authors

Solve-RD consortium authors
