# Supplementary material for: Solving unsolved rare neurological diseases—a Solve-RD viewpoint
Source: Eur J Hum Genet. 2021 May 10;29(9):1332–6. doi: 10.1038/s41431-021-00901-1 (PMC8440537; doi:10.1038/s41431-021-00901-1)
Supplement: Supplementary file 1 — Solve-RD Data Interpretation Taskforce ERN-RND authors [file 41431_2021_901_MOESM1_ESM.pdf]

Solve-RD Data Interpretation Taskforce ERN-RND (DITF-RND) author list

Jonathan Baets[1,2,3], Peter Balicza[4], Patrick Chinnery[5], Alexandra Dürr[6,7,8], Tobias Haack[9], Holger Hengel[10,11], Rita Horvath[12], Henry Houlden[13], Erik-Jan Kamsteeg[14], Christoph Kamsteeg[14], Katja Lohmann[15], Alfons Macaya[16], Anna Marcé-Grau[16], Ales Maver[17], Judit Molnar[4], Alexander Münchau[15], Borut Peterlin[17], Olaf Riess[9,18], Ludger Schöls[10,11], Rebecca Schüle*[10,11], Giovanni Stevanin[6,7,8,19,20], Matthis Synofzik*[10,11], Vincent Timmerman[21,22], Bart van de Warrenburg[23], Nienke van Os[23,24], Jana Vandrovcova[13], Melanie Wayand[10,11] and Carlo Wilke[10,11]
* indicates DITF leads.

Affiliations

[1]Peripheral Neuropathy Research Group, University of Antwerp, Antwerp, Belgium.
[2]Neuromuscular Reference Centre, Department of Neurology, Antwerp University Hospital, Antwerpen, Belgium.
[3]Laboratory of Neuromuscular Pathology, Institute Born-Bunge, University of Antwerp, Antwerpen, Belgium.
[4]Semelweis University Budapest, Hungary
[5]Center for Hereditary Tumor Syndromes, University Hospital Bonn, Bonn, Germany.
[6]Institut National de la Santé et de la Recherche Medicale (INSERM) U1127, Paris, France.
[7]Centre National de la Recherche Scientifique, Unité Mixte de Recherche (UMR) 7225, Paris, France.
[8]Unité Mixte de Recherche en Santé 1127, Université Pierre et Marie Curie (Paris 06), Sorbonne Universités, Paris, France.
[9]Institute of Medical Genetics and Applied Genomics, University of Tübingen, Tübingen, Germany.
[10]Department of Neurodegeneration, Hertie Institute for Clinical Brain Research (HIH), University of Tübingen, Tübingen, Germany.
[11]German Center for Neurodegenerative Diseases (DZNE), Tübingen, Germany.
[12] University of Cambridge, England, United Kingdom
[13] Department of Neuromuscular Diseases, UCL Queen Square Institute of Neurology and The National Hospital for Neurology and Neurosurgery, London, UK.
[14]Department of Human Genetics, Radboud University Medical Center, Nijmegen, The Netherlands.
[15]University of Lübeck, Lübeck, Germany
[16]Hospital Vall d'Hebron, Barcelona, Spain
[17]University of Ljubljana, Slovenia
[18]Centre for Rare Diseases, University of Tübingen, Tübingen, Germany.
[19]Institut du Cerveau -ICM, Paris, France.
[20]Ecole Pratique des Hautes Etudes, Paris Sciences et Lettres Research University, Paris, France.
[21]Peripheral Neuropathy Research Group, Department of Biomedical Sciences, University of Antwerp, Antwerp, Belgium.
[22]Institute Born Bunge, Antwerp, Belgium.
[23]Donders Institute for Brain, Cognition and Behaviour, Radboud University Medical Center, Nijmegen, The Netherlands.
[24]Department of Neurology, Radboud University Medical Center, Nijmegen, The Netherlands.
