# Supplementary material for: Solving unsolved rare neurological diseases—a Solve-RD viewpoint
Source: Eur J Hum Genet. 2021 May 10;29(9):1332–6. doi: 10.1038/s41431-021-00901-1 (PMC8440537; doi:10.1038/s41431-021-00901-1)
Supplement: Supplementary file 2 — Solve-RD consortium authors [file 41431_2021_901_MOESM2_ESM.pdf]

SolveRD

Solve-RD consortium* author list

Olaf Riess[1,2], Tobias B. Haack[1] , Holm Graessner[1,2], Birte Zurek[1,2], Kornelia Ellwanger[1,2], Stephan Ossowski[1], German Demidov[1], Marc Sturm[1], Julia M. Schulze-Hentrich[1], Rebecca Schüle[3,4], Christoph Kessler[3,4] , Melanie Wayand[3,4], Matthis Synofzik[3,4], Carlo Wilke[3,4], Andreas Traschütz[3,4], Ludger Schöls[3,4], Holger Hengel[3,4], Peter Heutink[3,4], Han Brunner[5,6,7], Hans Scheffer[5,6], Nicoline Hoogerbrugge[5,8] , Alexander Hoischen[5,8,9], Peter A.C. 't Hoen[8,10] ,Lisenka E.L.M. Vissers[5,7], Christian Gilissen[5,8], Wouter Steyaert[5,8], Karolis Sablauskas[5], Richarda M. de Voer[5,8], Erik-Jan Kamsteeg[5], Bart van de Warrenburg[7,11], Nienke van Os[7,11] , Iris te Paske[5,8], Erik Janssen[5,8], Elke de Boer[5,7], Marloes Steehouwer[5], Burcu Yaldiz[5], Tjitske Kleefstra[5,7], Anthony J. Brookes[12], Colin Veal[12], Spencer Gibson[12], Marc Wadsley[12], Mehdi Mehtarizadeh[12], Umar Riaz[12], Greg Warren[12], Farid Yavari Dizjikan[12], Thomas Shorter[12,] Ana Töpf[13], Volker Straub[13], Chiara Marini Bettolo[13], Sabine Specht[13], Jill Clayton-Smith[14], Siddharth Banka[14,15], Elizabeth Alexander[14], Adam Jackson[14], Laurence Faivre[16,17,18,19,20] Christel Thauvin[17,18,19,20,20], Antonio Vitobello[18], Anne-Sophie Denommé-Pichon[18], Yannis Duffourd[18,19], Emilie Tisserant[18], Ange-Line Bruel[18], Christine Peyron[21,22], Aurore Pélissier[22,22], Sergi Beltran[23,24], Ivo Glynne Gut[24,24], Steven Laurie[24], Davide Piscia[24], Leslie Matalonga[24], Anastasios Papakonstantinou[24], Gemma Bullich[24], Alberto Corvo[24], Carles Garcia[24], Marcos Fernandez-Callejo[24], Carles Hernández[24], Daniel Picó[24], Ida Paramonov[24], Hanns Lochmüller[24], Gulcin Gumus[25], Virginie Bros-Facer[26], Ana Rath[27], Marc Hanauer[27], Annie Olry[27], David Lagorce[27], Svitlana Havrylenko[27], Katia Izem[27], Fanny Rigour[27], Giovanni Stevanin[28,29,30,31,32], Alexandra Durr[29,30,31, 31,33], Claire-Sophie Davoine[29,30,31,32,32], Léna Guillot-Noel[29,30,31,32,32], Anna Heinzmann[29,30,31,31,34], Giulia Coarelli[29,30,31,31,34], Gisèle Bonne[35], Teresinha Evangelista[35], Valérie Allamand[35], Isabelle Nelson[35], Rabah Ben Yaou[35,36,37], Corinne Metay[35,38], Bruno Eymard[35,36], Enzo Cohen[35], Antonio Atalaia[35], Tanya Stojkovic[35,36], Milan Macek Jr.[39], Marek Turnovec[39], Dana Thomasová[39], Radka Pourová Kremliková[39], Vera Franková[39], Markéta Havlovicová[39], Vlastimil Kremlik[39], Helen Parkinson[40], Thomas Keane[40], Dylan Spalding[40], Alexander Senf[40], Peter Robinson[41], Daniel Danis[41], Glenn Robert[42], Alessia Costa[4242], Christine Patch[4242,43], Mike Hanna[44], Henry Houlden[45], Mary Reilly[44], Jana Vandrovcova[45], Francesco Muntoni[46,47], Irina Zaharieva[46], Anna Sarkozy[46], Vincent Timmerman[48,49], Jonathan Baets[50,51,52], Liedewei Van de Vondel[49,50], Danique Beijer[49,50], Peter de Jonghe[49,51], Vincenzo Nigro[53,54], Sandro Banfi[53,54], Annalaura Torella[53], Francesco Musacchia[53,54], Giulio Piluso[53], Alessandra Ferlini[55], Rita Selvatici[55], Rachele Rossi[55], Marcella Neri[55], Stefan Aretz[56,57], Isabel Spier[56,57], Anna Katharina Sommer[56], Sophia Peters[56], Carla Oliveira[58,59,60], Jose Garcia Pelaez[58,59], Ana Rita Matos[58,59], Celina São José[58,59], Marta Ferreira[58,59], Irene Gullo[58,59,60], Susana Fernandes[58,61], Luzia Garrido[62], Pedro Ferreira[58,59,63], Fátima Carneiro[58,59,60], Morris A. Swertz[64], Lennart Johansson[64], Joeri K. van der Velde[64], Gerben van der Vries[64], Pieter B. Neerincx[64], Dieuwke Roelofs-Prins[64], Sebastian Köhler[65], Alison Metcalfe[42,66], Alain Verloes[67,68], Séverine Drunat[67,68], Caroline Rooryck[69], Aurelien Trimouille[70], Raffaele Castello[54], Manuela Morleo[54], Michele Pinelli[54], Alessandra Varavallo[54], Manuel Posada De la Paz[71], Eva Bermejo Sánchez[71], Estrella López Martín[71], Beatriz Martínez Delgado[71], F. Javier Alonso García de la Rosa[71], Andrea Ciolfi[72], Bruno Dallapiccola[72], Simone Pizzi[72], Francesca Clementina Radio[72], Marco Tartaglia[72], Alessandra Renieri[73,74,75], Elisa Benetti[73], Peter Balicza[76], Maria Judit Molnar[76], Ales Maver[77], Borut Peterlin[77], Alexander Münchau[78], Katja Lohmann[78], Rebecca Herzog[78], Martje Pauly[78], Alfons Macaya[79], Anna Marcé-Grau[79], Andres Nascimiento Osorio[80], Daniel Natera de Benito[80], Hanns Lochmüller[81,82,83], Rachel Thompson[81,83], Kiran Polavarapu[81], David Beeson[84], Judith Cossins[84], Pedro M. Rodriguez Cruz[84], Peter Hackman[85], Mridul Johari[85], Marco Savarese[85], Bjarne Udd[85,86,87], Rita Horvath[88], Gabriel Capella[89], Laura Valle[89], Elke Holinski-Feder[90], Andreas Laner[90], Verena Steinke-Lange[90], Evelin Schröck[91], Andreas Rump[91,92],

[1] Institute of Medical Genetics and Applied Genomics, University of Tübingen, Tübingen, Germany.
[2] Centre for Rare Diseases, University of Tübingen, Tübingen, Germany.
[3] Department of Neurodegeneration, Hertie Institute for Clinical Brain Research (HIH), University of Tübingen, Tübingen, Germany.
[4] German Center for Neurodegenerative Diseases (DZNE), Tübingen, Germany.
[5] Department of Human Genetics, Radboud University Medical Center, Nijmegen, The Netherlands.
[6] Department of Clinical Genetics, Maastricht University Medical Centre, Maastricht, The Netherlands.
[7] Donders Institute for Brain, Cognition and Behaviour, Radboud University Medical Center, Nijmegen, The Netherlands.
[8] Radboud Institute for Molecular Life Sciences, Nijmegen, the Netherlands.
[9] Department of Internal Medicine and Radboud Center for Infectious Diseases (RCI), Radboud University Medical Center, Nijmegen, The Netherlands.

[10] Center for Molecular and Biomolecular Informatics, Radboud university medical center, Nijmegen, The Netherlands.
[11] Department of Neurology, Radboud University Medical Center, Nijmegen, The Netherlands.
[12] Department of Genetics and Genome Biology, University of Leicester, Leicester, UK.
[13] John Walton Muscular Dystrophy Research Centre, Translational and Clinical Research Institute, Newcastle University and Newcastle Hospitals NHS Foundation Trust, Newcastle upon Tyne, UK.
[14] Division of Evolution and Genomic Sciences, School of Biological Sciences, Faculty of Biology, Medicine and Health, University of Manchester, Manchester M13 9WL, UK.
[15] Manchester Centre for Genomic Medicine, St Mary's Hospital, Manchester University Hospitals NHS Foundation Trust, Health Innovation Manchester, Manchester M13 9WL, UK.
[16] Dijon University Hospital, Genetics Department, Dijon, France.
[17] Dijon University Hospital, Centre of Reference for Rare Diseases: Development disorders and malformation syndromes, Dijon, France.
[18] Inserm - University of Burgundy-Franche Comté, UMR1231 GAD, Dijon, France.
[19] Dijon University Hospital, FHU-TRANSLAD, Dijon, France.
[20] Dijon University Hospital, GIMI institute, Dijon, France.
[21] University of Burgundy-Franche Comté, Dijon Economics Laboratory, Dijon, France.
[22] University of Burgundy-Franche Comté, FHU-TRANSLAD, Dijon, France.
[23] CNAG-CRG, Centre for Genomic Regulation (CRG), The Barcelona Institute of Science and Technology, Baldiri Reixac 4, Barcelona 08028, Spain.
[24] Universitat Pompeu Fabra (UPF), Barcelona, Spain.
[25] EURORDIS-Rare Diseases Europe, Sant Antoni Maria Claret 167 - 08025 Barcelona, Spain.
[26] EURORDIS-Rare Diseases Europe, Plateforme Maladies Rares, 75014 Paris, France.
[27] INSERM, US14 - Orphanet, Plateforme Maladies Rares, 75014 Paris, France.
[28] Institut National de la Santé et de la Recherche Medicale (INSERM) U1127, Paris, France.
[29] Centre National de la Recherche Scientifique, Unité Mixte de Recherche (UMR) 7225, Paris, France.
[30] Unité Mixte de Recherche en Santé 1127, Université Pierre et Marie Curie (Paris 06), Sorbonne Universités, Paris, France.
[31] Institut du Cerveau -ICM, Paris, France.
[32] Ecole Pratique des Hautes Etudes, Paris Sciences et Lettres Research University, Paris, France.
[33] Centre de Référence de Neurogénétique, Hôpital de la Pitié-Salpêtrière, Assistance Publique-Hôpitaux de Paris (AP-HP), Paris, France.
[34] Hôpital de la Pitié-Salpêtrière, Assistance Publique-Hôpitaux de Paris (AP-HP), Paris, France.
[35] Sorbonne Université, INSERM UMRS_974, Center of Research in Myology, 75013 Paris, France.
[36] AP-HP, Centre de Référence de Pathologie Neuromusculaire Nord, Est, Ile-de-France, Institut de Myologie, G.H. Pitié-Salpêtrière, F-75013 Paris, France.
[37] Institut de Myologie, Equipe Bases de données, G.H. Pitié-Salpêtrière, F-75013 Paris, France.
[38] AP-HP, Unité Fonctionnelle de Cardiogénétique et Myogénétique Moléculaire et Cellulaire, G.H. Pitié-Salpêtrière, F-75013 Paris, France.
[39] Department of Biology and Medical Genetics, Charles University Prague-2nd Faculty of Medicine and University Hospital Motol, Prague, Czech Republic.
[40] European Bioinformatics Institute, European Molecular Biology Laboratory, Wellcome Genome Campus, Hinxton, Cambridge, United Kingdom.
[41] Jackson Laboratory for Genomic Medicine, Farmington, CT 06032, USA.
[42] Florence Nightingale Faculty of Nursing and Midwifery, King's College, London, UK.
[43] Genetic Counselling, Genomics England, Queen Mary University of London, Dawson Hall, EC1M 6BQ, London.
[44] MRC Centre for Neuromuscular Diseases and National Hospital for Neurology and Neurosurgery, UCL Queen Square Institute of Neurology, London, UK.
[45] Department of Neuromuscular Diseases, UCL Queen Square Institute of Neurology, London, UK.
[46] Dubowitz Neuromuscular Centre, UCL Great Ormond Street Hospital, London, UK.
[47] NIHR Great Ormond Street Hospital Biomedical Research Centre, London, United Kingdom.
[48] Peripheral Neuropathy Research Group, Department of Biomedical Sciences, University of Antwerp, Antwerp, Belgium.
[49] Institute Born Bunge, Antwerp, Belgium.
[50] Peripheral Neuropathy Research Group, University of Antwerp, Antwerp, Belgium.
[51] Neuromuscular Reference Centre, Department of Neurology, Antwerp University Hospital, Antwerpen, Belgium.
[52] Laboratory of Neuromuscular Pathology, Institute Born-Bunge, University of Antwerp, Antwerpen, Belgium.

[53] Dipartimento di Medicina di Precisione, Università degli Studi della Campania "Luigi Vanvitelli," Napoli, Italy.
[54] Telethon Institute of Genetics and Medicine, Pozzuoli, Italy.
[55] Unit of Medical Genetics, Department of Medical Sciences, University of Ferrara, Italy.
[56] Institute of Human Genetics, University of Bonn, Bonn, Germany.
[57] Center for Hereditary Tumor Syndromes, University Hospital Bonn, Bonn, Germany.
[58] i3S - Instituto de Investigação e Inovação em Saúde, Universidade do Porto, Portugal.
[59] IPATIMUP - Institute of Molecular Pathology and Immunology of the University of Porto, Portugal.
[60] Departament of Pathology, Faculty of Medicine, University of Porto, Portugal.
[61] Departament of Genetics, Faculty of Medicine, University of Porto, Portugal.
[62] CHUSJ, Centro Hospitalar e Universitário de São João, Porto, Portugal.
[63] Faculty of Sciences, University of Porto, Portugal.
[64] Department of Genetics, Genomics Coordination Center, University Medical Center Groningen, University of Groningen, Groningen, The Netherlands.
[65] NeuroCure Cluster of Excellence, Charité Universitätsklinikum, Charitéplatz 1, 10117 Berlin, Germany.
[66] College of Health, Well-being and Life-Sciences, Sheffield Hallam University, Sheffield, UK.
[67] Dept of Genetics, Assistance Publique-Hôpitaux de Paris - Université de Paris, Robert DEBRE University Hospital, 48 bd SERURIER, Paris, France.
[68] INSERM UMR 1141 "NeuroDiderot", Hôpital R DEBRE, Paris, France.
[69] Univ. Bordeaux, MRGM INSERM U1211, CHU de Bordeaux, Service de Génétique Médicale , F-33000 Bordeaux, France.
[70] Laboratoire de Génétique Moléculaire, Service de Génétique Médicale, CHU Bordeaux – Hôpital Pellegrin, Place Amélie Raba Léon, 33076 Bordeaux Cedex, France.
[71] Institute of Rare Diseases Research, Spanish Undiagnosed Rare Diseases Cases Program (SpainUDP) & Undiagnosed Diseases Network International (UDNI), Instituto de Salud Carlos III, Madrid, Spain.
[72] Genetics and Rare Diseases Research Division, Ospedale Pediatrico Bambino Gesù, IRCCS, 00146 Rome, Italy.
[73] Med Biotech Hub and Competence Center, Department of Medical Biotechnologies, University of Siena, Italy.
[74] Medical Genetics, University of Siena, Italy.
[75] Genetica Medica, Azienda Ospedaliero-Universitaria Senese, Italy.
[76] Institute of Genomic Medicine and Rare Diseases, Semmelweis University, Budapest, Hungary.
[77] Clinical institute of genomic medicine, University medical centre Ljubljana, Slovenia.
[78] Institute of Neurogenetics, University of Lübeck, Lübeck, Germany.
[79] Neurology Research Group, Vall d'Hebron Research Institute, Universitat Autònoma de Barcelona, Barcelona, Spain.
[80] Neuromuscular Disorders Unit , Department of Pediatric Neurology. Hospital Sant Joan de Déu, Barcelona, Spain.
[81] Department of Neuropediatrics and Muscle Disorders, Medical Center, Faculty of Medicine, University of Freiburg, Freiburg, Germany.
[82] Centro Nacional de Análisis Genómico (CNAG-CRG), Center for Genomic Regulation, Barcelona Institute of Science and Technology (BIST), Barcelona, Spain.
[83] Children's Hospital of Eastern Ontario Research Institute, University of Ottawa, Ottawa, ON, Canada.
[84] Nuffield Department of Clinical Neurosciences, University of Oxford, UK.
[85] Folkhälsan Research Centre and Medicum, University of Helsinki, Helsinki, Finland.
[86] Tampere Neuromuscular Center, Tampere, Finland.
[87] Vasa Central Hospital, Vaasa, Finland.
[88] Department of Clinical Neurosciences, University of Cambridge, Cambridge, UK.
[89] Bellvitge Biomedical Research Institute (IDIBELL), Barcelona, Spain.
[90] Medical Genetics Center (MGZ), Munich, Germany.
[91] Institute for Clinical Genetics, Faculty of Medicine Carl Gustav Carus, Technical University Dresden, Dresden, Germany.
[92] Center for Personalized Oncology, University Hospital Carl Gustav Carus, Technical University Dresden, Dresden, Germany.
